# Effects of Drug-Free Pectin Hydrogel Films on Thermal Burn Wounds in Streptozotocin-Induced Diabetic Rats

**DOI:** 10.3390/polym14142873

**Published:** 2022-07-15

**Authors:** Nur Nadhirah Nordin, Nur Karimah Aziz, Idanawati Naharudin, Nor Khaizan Anuar

**Affiliations:** 1 Faculty of Pharmacy, Universiti Teknologi MARA, Puncak Alam 42300, Selangor, Malaysia; nadhirahnordin13@gmail.com (N.N.N.); kimah_1307@yahoo.com (N.K.A.); idana482@uitm.edu.my (I.N.); 2 Non-Destructive Biomedical and Pharmaceutical Research Centre, Smart Manufacturing Research Institute, Universiti Teknologi MARA, Puncak Alam 42300, Selangor, Malaysia; 3 Food Process and Engineering Research Group (FOPERG), Universiti Teknologi MARA, Shah Alam 40450, Selangor, Malaysia

**Keywords:** drug-free, pectin, hydrogel, wound healing, diabetic rats

## Abstract

This study aims to examine the influence of drug-free pectin hydrogel films on partial-thickness burn wounds using streptozotocin-induced diabetic rats as the animal model. Thirty male Sprague Dawley rats were included in the wound healing study, and scalding water was used to produce wounds in the dorsum region of the rats. Two different formulations of pectin hydrogel films, PH 2.5% and PH 5%, were prepared using a solvent evaporation method. MEBO^®^ (moist exposed burn ointment), a commercial herbal formulation was used as a positive control. The progress of the wound healing was observed and compared between untreated normal rats, untreated diabetic rats, diabetic rats treated with MEBO^®^, diabetic rats treated with PH 2.5%, and diabetic rats treated with PH 5%. The results showed that diabetic rats treated with PH 5% healed faster than the untreated diabetic rats and diabetic rats treated with PH 2.5%. Interestingly, the diabetic rats treated with PH 5% healed as well as diabetic rats treated with MEBO^®^, where wounds were healed entirely on day 20. Nevertheless, both PH 2.5% and PH 5% showed a greater zone of inhibition than MEBO^®^ when tested against *Staphylococcus aureus*.

## 1. Introduction

According to the International Diabetes Federation, about 537 million people, or 1 in 10 adults (20 to 79 years), were living with diabetes in 2021. The number is alarmingly expected to increase to 783 million by 2045 [1]. Diabetes mellitus is one of the main causes of delayed wound healing involving multiple complex pathophysiological mechanisms [2,3,4]. Uncontrolled cellular functions such as defects in phagocytosis and bactericidal capacity, defective T cell immunity, as well as dysfunction of fibroblasts and epidermal cells are usually associated with diabetic wounds [3]. The prolonged wound healing in diabetic patients can also lead to diabetic foot ulcers [5,6]. The management of burn wounds in patients who have poor wound healing progress is one of the greatest problems in therapeutic treatment [7]. Based on a retrospective review by Low et al. [8], burn patients with diabetes have a higher risk of wound infections and longer lengths of stay in health facilities compared to non-diabetic patients.

Polymeric dressing materials such as foams, films, hydrocolloids, and alginates are currently being used as wound dressings [9]. However, these materials have limitations as wound dressings. For example, some films are non-absorbent allowing the accumulation of wound exudates, foams are an opaque layer which cause difficulties in wound monitoring, and hydrocolloids have a high leakage of exudates [9].

Pectin is a component of plant cell walls which consists of mostly galacturonic acids with their carboxyl moieties partly esterified with methoxy groups [10]. It can be formulated into hydrogels for the entrapment and delivery of drugs, proteins, and cells [11]. The amorphous nature of pectin makes the polymer ideal to be used in skin tissue applications [12]. The hydrophilic part of pectin reacts with wound exudates and develops a soft gel, which is responsible for the elimination and control of wound exudates [13]. The high hydrophilicity of pectin is similar to that of other wound healing polyanions, and it has been reported to have strong anti-inflammatory effects, making it desirable for the treatment of burns and chronic diabetic wounds [14].

Hydrogel wound dressings have several features that make them ideal wound dressings, such as good self-healing properties and excellent deformability [15]. Hydrogel can absorb and retain wound exudates, which promotes fibroblast proliferation and keratinocyte migration [9,12]. Its structure can prevent wound infections and facilitate the transport of bioactive molecules (antibiotics) and pharmaceuticals to the wound centre [9]. The significant tissue-like water content provides the flexibility and elasticity needed to adapt to wounds located in different body sites. It is also a type of wound dressing that does not need to be changed regularly and will therefore not interfere with the tissue repair process as there is no painful “ripping open” of the wound [16]. Therefore, this study aims to investigate the ability of drug-free pectin hydrogel films to promote wound healing in partial-thickness burn wounds using diabetic-induced rats as the injury model.

## 2. Materials and Methods

### 2.1. Materials

MEBO^®^ (moist exposed burn ointment), a herbal formulation containing b-sitosterol, baicalin, and berberine as the active ingredients in a base of beeswax and sesame oil, was purchased from Triniaire Sdn. Bhd, Malaysia. Tiletamine/Zolazepam (Zoletil 50) (Virbac, Mumbai, India) was used as an anaesthetic agent. Pentobarbital (Dolethal) was obtained from Vetoquinol (Lure Cedex, France). Pectin (methoxyl content = 9.0%, galacturonic acid content = 87.6%) was purchased from Sigma Aldrich (St. Louis, MO, USA). Streptozotocin (STZ) and citrate buffer (pH = 4.5) were provided by Sigma Aldrich (St. Louis, MO, USA). Glycerol and ethanol were purchased from Sigma Aldrich (Saint-Quentin Fallavier, France). Glutaraldehyde (Grade II, 25% in water) was obtained from Sigma Aldrich (St. Louis, MO, USA). Formalin solution was procured from Fisher Chemical (Loughborough, UK). Paraffin was purchased from Leica Biosystems (Newcastle, UK). Staining reagents, such as haematoxylin, were acquired from VWR International (Lutterworth, UK), and eosin was obtained from Microm International (Walldorf, Germany). Deionised water was used throughout the experiment.

### 2.2. Preparation of Pectin Hydrogel Films

The pectin hydrogel films were prepared using a solvent evaporation method. Pectin (2.5% *w*/*w*) was dissolved in deionised water under constant stirring at 600 rpm and 60 °C for 10 min, followed by continuous stirring at room temperature (25 ± 2 °C) for 24 h to obtain a homogenous solution. In sequence, 0.8% (*w*/*w*) glutaraldehyde and 0.25% (*w*/*w*) glycerol were added to the pectin solution and were stirred for another 40 min, respectively. The mixture was then poured into a glass Petri dish (internal diameter = 3 cm) and kept in an oven at 40 ± 0.5 °C for 24 h. The formed films were soaked in 3 mL of ethanol in a glass lid-covered Petri dish for 1 h to remove the unreacted glutaraldehyde and subsequently dried for 6 h at 40 ± 0.5 °C. The steps were repeated for 5% (*w*/*w*) pectin and all other experimental conditions were maintained.

### 2.3. Differential Scanning Calorimetry (DSC)

Three mg of pectin hydrogel film fragments were filled in a standard aluminium pan and heated at a heating rate of 10 °C/min from 30 to 380 °C using a differential scanning calorimeter (Pyris 6 DSC, Perkin Elmer, Waltham, MA, USA). Constant purging of nitrogen at 40 mL/min was used throughout the study. The experiment was conducted in triplicate, and the results were averaged.

### 2.4. Fourier Transform Infrared Spectroscopy (FTIR)

The 2.5% (*w*/*w*) of pectin hydrogel film fragments were mixed with potassium bromide (KBr, Sigma Aldrich, Schnelldorf, Germany) using an agate pestle and mortar set. The mixture was compacted into a disc and scanned between a wavenumber of 450 to 4000 cm^−1^ at a resolution of 4 cm^−1^ using an FTIR spectrometer (Spectrum RX1 FTIR system, Perkin-Elmer, Waltham, MA, USA). The experiment was conducted in triplicate, and the results were averaged.

### 2.5. Experimental Animals

Thirty male Sprague Dawley rats aged two months and weighing 250–300 g were obtained from the Laboratory Animal Facility and Management (LAFAM), Universiti Teknologi MARA, Malaysia. The rats were caged individually after burn wound induction until the completion of wound healing. The animals were given free access to deionised water and standard pelletised food. The room temperature was set at 25 ± 2 °C and relative humidity was maintained at 65 ± 5%. All rats were randomly divided into five groups of six animals each:

Group 1: untreated normal rats.
Group 2: untreated diabetic rats (negative control).
Group 3: diabetic rats treated with MEBO^®^ (positive control).
Group 4: diabetic rats treated with 2.5% (*w*/*w*) pectin hydrogel (PH 2.5%).
Group 5: diabetic rats treated with 5% (*w*/*w*) pectin hydrogel (PH 5%).

The experimental protocol was approved by the Committee on Animal Research and Ethics of the Universiti Teknologi MARA (UiTM CARE: 270/2019 (15 February 2019)).

### 2.6. Induction of Diabetes Mellitus

Twenty-four rats were induced with a single dose (60 mg/kg) of freshly prepared streptozotocin (STZ; Sigma Aldrich (St. Louis, MO, USA) administered through the intraperitoneal cavity. The rats were given a 5% glucose solution as drinking water following the STZ injection to prevent hypoglycaemia [17]. Fasting blood glucose levels were measured using a glucometer in blood samples obtained from a tail vein 72 h after the injection of STZ. Rats with blood glucose levels above 200 mg/dL were defined as diabetic [18].

### 2.7. In Vivo Wound Healing Study

Thirty healthy Sprague Dawley rats (male; 250–300 g) were randomly divided into five groups of six animals each. Tiletamine/Zolazepam (Zoletil) was used to anesthetise the rats via the intraperitoneal cavity at 40 mg/kg body weight of rats, respectively. The dorsal hair of the rats was trimmed using a shaver and was cleaned with an alcohol swab. A rounded plastic ring (internal diameter = 1.27 cm; external diameter = 2.20 cm; height = 5 cm) was attached to the trimmed area of rats. Subsequently, the plastic ring was filled with 6 mL of scalding deionised water (65 ± 5 °C) for 1 min in 9 repetitive cycles [19]. The pectin hydrogel was applied to the wounded skin which was then covered with a standard gauze and adhesive tape. Meanwhile, the wounded skin of the rats in the negative control group was covered with standard gauze and adhesive tape only. The wounded skin of rats in the positive control group was applied with MEBO^®^ and covered with standard gauze and adhesive tape. The macroscopic images of wounds were digitally photographed, and the wound diameter was measured using a digital calliper (Mitutoyo, Sakado, Japan) on days 0, 2, 7, 14, and 20. The wound size was calculated using the area of a circle formula. The percentage of re-epithelialisation was calculated based on wound size [19], as shown in the following equation:

percentage of re-epithelialisation = (W_1_ − W_2_)/W_1_ × 100% (1)

where W_1_ is the wound size at *t* = 0 and W_2_ is the wound at a particular time interval, *t*.

### 2.8. Histological Study

Rats were euthanised on days 0 and 20 by the injection of 0.25 mL of phenobarbital (Dolethal) (200 mg/mL) through the intraperitoneal cavity. The wounded skin was surgically removed, then fixed in 10% formalin solution (Fisher Chemical, Loughborough, UK) and subsequently embedded in paraffin (Leica, Newcastle, UK). Tissue sections of 5 µm thickness were sliced and stained with haematoxylin and eosin (H&E). A bright-field microscope (DM2000, Leica, Wetzlar, Germany) with a magnification level of 200× was used to view the stained skin, and the tissue images were captured.

### 2.9. Antimicrobial Activity

The culture of *S. aureus* was inoculated in nutrient broth, incubated at 37 °C for 18–20 h and was used for its resistant/susceptibility test against pectin hydrogel films. The evaluation of the pectin hydrogel films was carried out using the Kirby–Bauer technique (disc diffusion method). Samples were cut into circular discs (6 mm in diameter). The Whatman Grade AA disc (6 mm) without a sample was set as the negative control and the disc soaked with MEBO^®^ was set as the positive control. All discs were sterilised by UV radiation for 15 min and subsequently pressed into the bacteria-seeded agar plates (2 per plate). The plates were incubated for 24 h at 37 °C and the zone of inhibition was measured using a digital calliper (Mitutoyo, Sakado, Japan). The zone of inhibition diameter was recorded as an average of at least three readings.

### 2.10. Statistical Analysis

The results were presented as a mean of at least three repetitions with the corresponding standard deviation. Statistical data analysis was carried out using SPSS software version 25.0 and a statistically significant difference was expressed by *p* < 0.05 on the Student’s *t*-test. Analysis of variance (ANOVA) and post hoc analysis using Tukey’s HSD test were used when essentially required.

## 3. Results and Discussion

**Figure 1:**
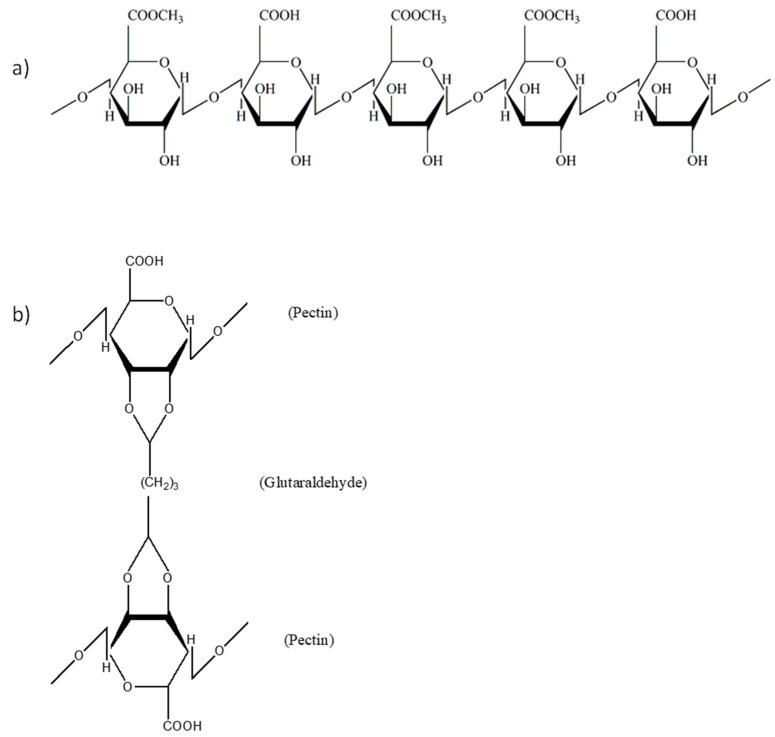
Chemical structure of (**a**) pectin and (**b**) pectin hydrogel film.

**Figure 2:**
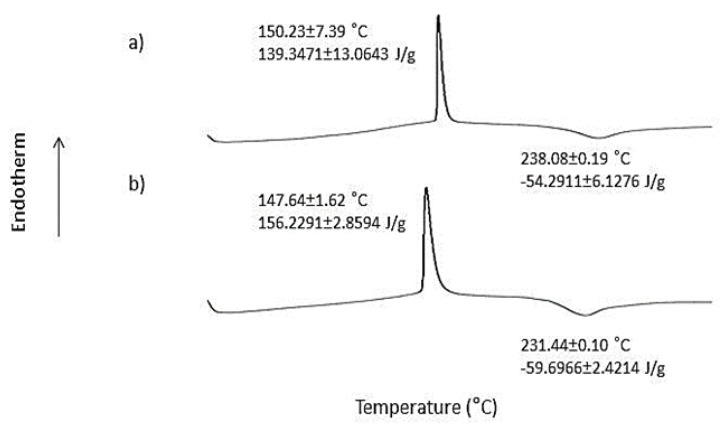
DSC thermograms of (**a**) unprocessed pectin and (**b**) pectin hydrogel film.

**Figure 3:**
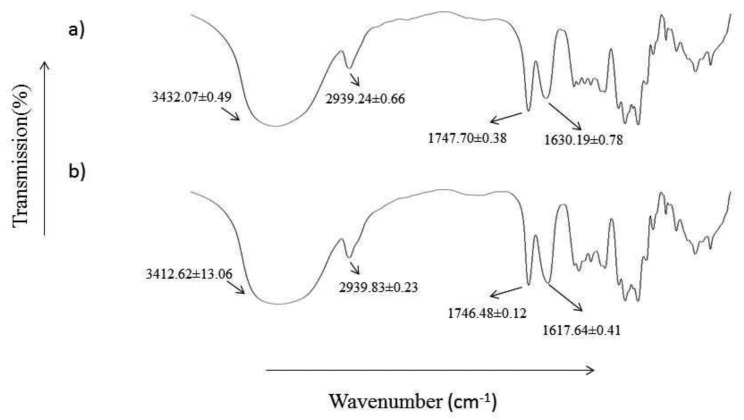
FTIR spectra of (**a**) unprocessed pectin and (**b**) pectin hydrogel film.

Pectin is an acidic complex polysaccharide abundant in the cell wall of plants [20,21]. Adding glutaraldehyde and glycerol into the pectin solution yielded a transparent pectin hydrogel film with a highly smooth and homogenous surface. Glutaraldehyde acted as a crosslinker, while glycerol was used as a plasticiser to improve the flexibility of the hydrogel film. Glutaraldehyde is an organic compound known as dialdehyde. Its aldehydic groups are highly reactive and have a great affinity for forming covalent bonds with chemical moieties such as phenols, hydroxyl, thiols, amines, and imidazoles [22]. Figure 1 illustrates the chemical structure of unprocessed pectin and pectin hydrogel, where pectin is cross-linked by glutaraldehyde via the hydroxyl groups. In addition, the DSC and FTIR analysis showed that pectin hydrogel films exhibited a greater extent of polymer-polymer interaction at O-H moiety when compared to the unprocessed pectin. This was indicated by the increase in endothermic enthalpy values from 139.35 ± 13.06 J/g of unprocessed pectin to 156.23 ± 2.86 J/g of pectin hydrogel (Figure 2), as well as the decrease in FTIR wavenumber corresponding to O-H at 3432.07 ± 0.49 cm^−1^ of unprocessed pectin to 3412.62 ± 13.06 cm^−1^ of pectin hydrogel (Figure 3). The physicochemical characteristics of pectin hydrogel film appeared suitable for use as a wound dressing and were further verified by the wound healing study.

**Figure 4:**
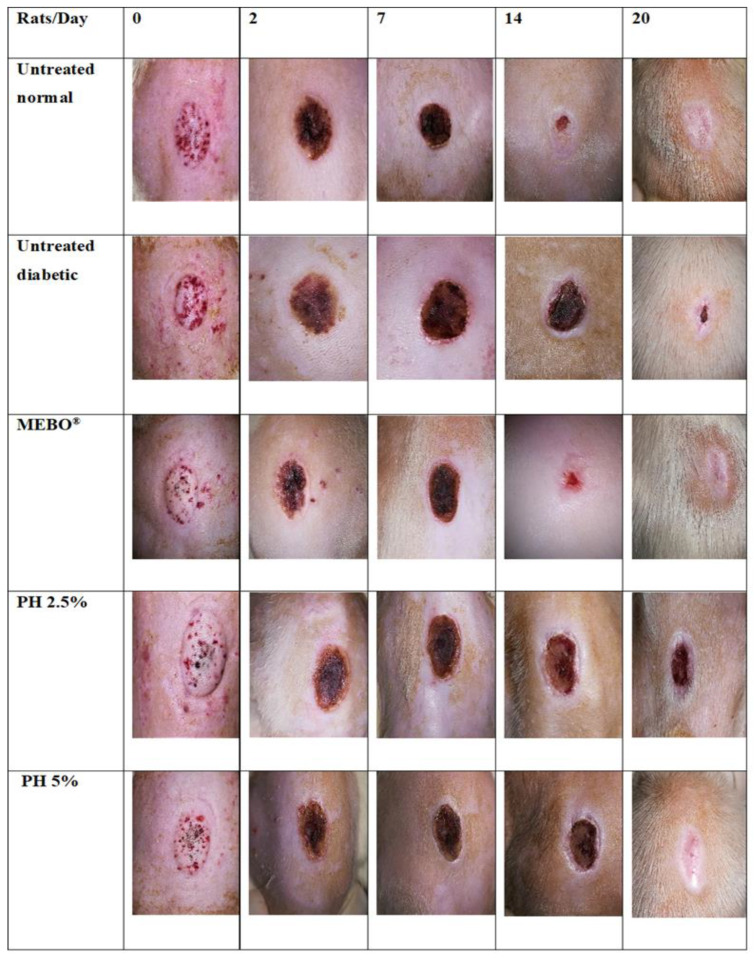
Photographs of wounds for various animal groups: untreated normal rats, untreated diabetic rats, and diabetic rats treated with MEBO^®^, PH 2.5%, and PH 5%.

Wound healing is a complex and dynamic biological process resulting in the restoration of tissue integrity [17]. For partial-thickness burn wounds, the skin has an ability to self-heal where some of the healthy dermis remains [18]. These burn wounds require an ideal wound dressing with multiple functions for maintaining a moist wound environment, providing protection from infections, removing wound exudates, and promoting wound healing [18]. A partial-thickness burn model was established for the evaluation of the present pectin hydrogel film dressing. The application of pectin hydrogel film has shown positive effects in accelerating the rate of wound healing. In untreated normal rats, the wound size was smaller (Student’s *t*-test: *p* = 0.27) and re-epithelialisation was higher (Student’s *t*-test: *p* = 0.55) than in untreated diabetic rats. The wound healing rate was slower in untreated diabetic rats due to the delayed wound repair. However, wound healing was improved with treatment in diabetic rats. The macroscopic images of burn wounds on days 0, 2, 7, 14, and 20 are shown in Figure 4.

**Figure 5:**
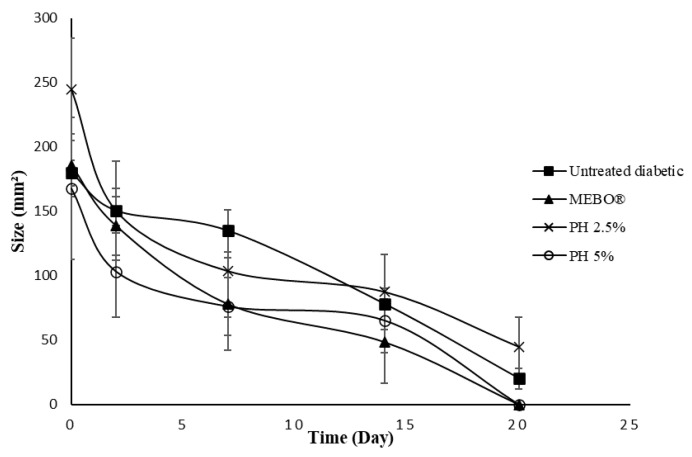
Profiles of wound size.

**Figure 6:**
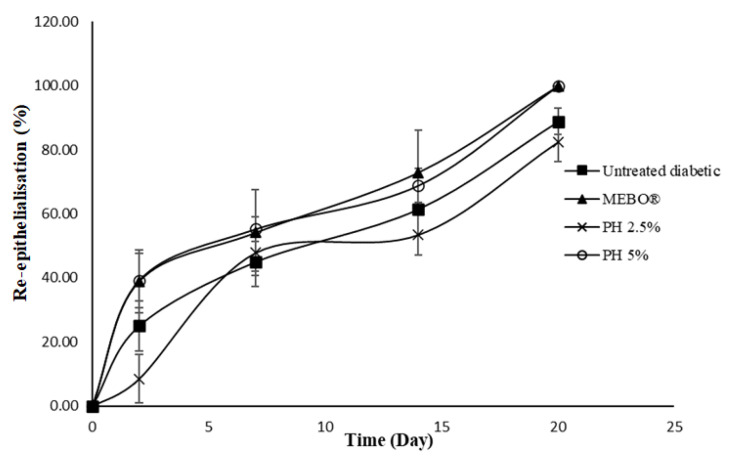
Rate of re-epithelialisation.

On day 0, following the induction of a burn wound by using scalding water, the skin developed a white eschar with a hyperaemic zone at the border of the wound (Figure 4). With time, the white eschar was transformed into a state of full hyperaemia which indicated there was an increase in blood flow to the wounded area [16]. A smaller wound size was noticed in rats treated with PH 5% than in untreated diabetic and diabetic rats treated with PH 2.5% within the first 2 days of treatment (Figure 4 and Figure 5; ANOVA: *p* = 0.15). The re-epithelialisation rate was higher in diabetic rats treated with PH 5% than in untreated diabetic rats on day 2 (Figure 6, ANOVA: *p* = 0.13). Additionally, the rats treated with PH 5% demonstrated a significantly higher re-epithelialisation propensity than PH 2.5% and MEBO^®^ on day 2 (Figure 6, ANOVA: *p* < 0.05). On day 7, scabs had formed over the wound of all groups. However, scabs covering the wound area in diabetic rats treated with MEBO^®^, PH 2.5%, and PH 5% were significantly smaller in size than in untreated diabetic rats (Figure 4 and Figure 5, ANOVA: *p* < 0.05). On day 14, the scabs formed in rats treated with MEBO^®^ had detached, while the scabs formed in other groups of rats were still intact.

It was noted that scabs were completely detached on day 20 for diabetic rats treated with MEBO^®^ and PH 5%; therefore, the wound size and re-epithelialisation propensity between both groups were similar (Figure 5 and Figure 6). According to Fonder et al. [23], hydrogel wound dressings can assist autolytic debridement by moisturising the dehydrated and non-viable tissues, thus promoting their detachment from healthy tissues. It is a less invasive method with a lower risk of bacterial infection than other types of wound debridement [24,25]. Nevertheless, there was a significantly larger wound size and a lower re-epithelialisation rate for rats treated with PH 2.5% than PH 5% (Figure 4, Figure 5 and Figure 6; ANOVA: *p* < 0.05). Pectin with a concentration of 5% (*w*/*w*) could be ideal for the current experimental settings, which can promote wound healing in diabetic-induced rats. This is supported by other studies by Valle et al. [26] and Rezvanian et al. [27]. They used a 5% (*w*/*w*) pectin solution in preparing the pectin–allantoin hydrogel and the alginate–pectin hydrogel film, respectively for the in vivo wound healing study using Wistar rats as the animal model. Another study by Kocaaga and co-workers [28] employed a lower concentration of pectin at 2% (*w*/*w*) to prepare pectin–zeolite-based wound dressings; nevertheless, the wound healing study was conducted in vitro. Hence, in future studies, it could be useful to examine the in vivo wound healing effects of pectin hydrogel film at a higher concentration than 5% (*w*/*w*).

In contrast to diabetic rats treated with MEBO^®^ and PH 5%, scabs were seen intact in the wounds of untreated diabetic rats and diabetic rats treated with PH 2.5% on day 20 (Figure 4). The diabetic rats treated with MEBO^®^ and PH 5% showed a remarkably faster wound size reduction and a higher re-epithelialisation than untreated diabetic rats and diabetic rats treated with PH 2.5% (Figure 5 and Figure 6). The structurally cross-linked hydrophilic hydrogels have been proven to provide good fluid absorbance, which is very useful for faster absorption of exudates during the wound healing process [9,29]. In addition, pectin hydrogel can provide excellent cell adhesion and proliferation as it is a cell instructive hydrogel [12,30].

**Figure 7:**
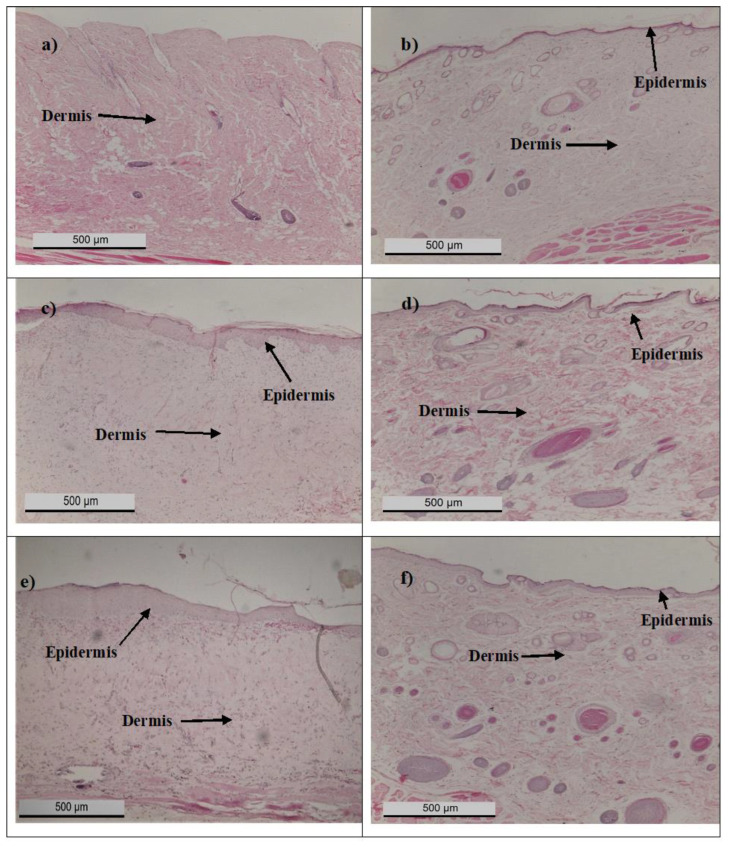
Photomicrographs of the H&E-stained histology section for (**a**) diabetic rats at day 0, (**b**) untreated normal rats at day 20, (**c**) untreated diabetic rats at day 20, (**d**) diabetic rats treated with MEBO^®^ at day 20, (**e**) diabetic rats treated with PH 2.5% at day 20, and (**f**) diabetic rats treated with PH 5% at day 20. Scale bar = 500 µm.

The histology study endorsed the characteristic of partial-thickness burn wounds. On day 0, the entire epidermis was destroyed, while some of the dermis remained in diabetic rats following the induction of burn wounds (Figure 7a). On day 20, the wounds of untreated normal rats (Figure 7b), as well as rats treated with MEBO^®^ (Figure 7d) and PH 5% (Figure 7f), were completely healed, as shown by the thickened epidermis on the wounds. However, it was observed that the epidermis was not fully formed in untreated diabetic rats (Figure 7c), and there was a thick scab on the epidermis of wounds treated with PH 2.5%, thus indicating non-complete wound healing (Figure 7e). These results suggested that PH 5% was able to promote skin regeneration in diabetic burn wounds by stimulating re-epithelialisation. Timely re-epithelialisation is important in burn injury since delayed wound closure will increase the risk of wound infection [31].

**Figure 8:**
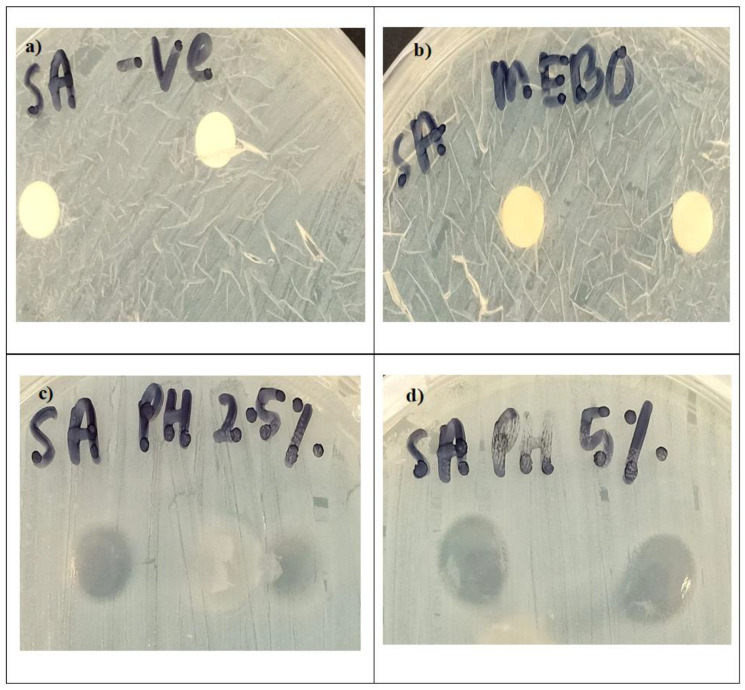
Antimicrobial activity of (**a**) negative control, (**b**) MEBO^®^, (**c**) PH 2.5%, and (**d**) PH 5% against *S. aureus* represented by the zone of inhibition.

Bacterial infection is one of the major causes of wound infection [32]. Therefore, the antimicrobial activity of PH 2.5%, PH 5%, and MEBO^®^ was examined. Delayed wound healing in diabetic patients can result in diabetic foot ulcers [5]. Diabetics with ulcers commonly have infections with Gram-positive organisms such as *S. aureus* and Gram-negative organisms such as *Pseudomonas aeruginosa* [33]. In this study, the antimicrobial activity of PH was investigated against *S. aureus*. Both PH 2.5% and PH 5% exhibited a comparatively greater zone of inhibition than MEBO^®^, with results of 0.61 ± 0.01, 0.77 ± 0.01, and 0.33 ± 0.01 mm, respectively (Figure 8). The results suggested that drug-free pectin hydrogel can protect wounds from infection and prevent microorganisms from reaching the wound area [8]. The antimicrobial activity of pectin, coupled with its ability to maintain an acidic environment, is expected to act as a barrier against microorganisms and limit their microbial activities [34,35].

## Data Availability

The authors confirm that the data supporting the findings of this study are available within the article.
